# Agrochemical residues and food safety: In-depth in silico assessment of the degradation mechanism of chlorpyrifos-oxon by niobium dioxide (NbO₂), a naturally occurring and cost-effective mineral

**DOI:** 10.1007/s00894-026-06865-7

**Published:** 2026-07-31

**Authors:** Letícia S. Braga, Adelia J. A. Aquino, Teodorico C. Ramalho

**Affiliations:** 1 Department of Chemistry, Universidade Federal de Lavras, Campus Universitário, Lavras-MG, 37200-000 Brazil; 2 Department of Mechanical and Aerospace Engineering, Texas Tech University, Lubbock, TX 79409-1021 USA; 3 Department of Chemistry, Faculty of Science, University of Hradec Kralove, Hradec Kralove, Czech Republic

**Keywords:** Chlorpyrifos-oxon, Niobium dioxide, Density functional theory, Food safety

## Abstract

**Context:**

The widespread use of chlorpyrifos-based pesticides has raised increasing concerns regarding environmental persistence, food safety, and human exposure to toxic organophosphate residues. In this work, the degradation mechanism of chlorpyrifos-oxon mediated by niobium dioxide (NbO₂), a naturally occurring and low-cost mineral, was investigated through in silico approaches. The results indicate that NbO₂ can promote the degradation of chlorpyrifos-oxon through energetically favorable pathways, reducing activation barriers and stabilizing key intermediates along the reaction coordinate. Electronic structure analyses revealed significant charge redistribution during the degradation process, supporting the catalytic role of the mineral surface. These findings reinforce the potential application of NbO₂ as an accessible material for agrochemical remediation and food safety strategies.

**Methods:**

Density functional theory calculations were performed using the ωB97X-D3 functional with the def2-TZVP basis set. Geometry optimizations, vibrational frequency analyses, intrinsic reaction coordinate calculations, and electronic structure analyses were carried out using ORCA software. Solvent effects were considered through an implicit solvation model to better represent the reaction environment.

**Supplementary Information:**

The online version contains supplementary material available at 10.1007/s00894-026-06865-7.

## Introduction

A study carried out by the United Nations estimates that the world population in 2024 will be over 8 billion people and, in 2050, over 9.5 billion [1]. Given these projections, it is difficult not to associate population growth with the increasing global demand for food. To meet this demand, agricultural production must expand. However, it is important to bear in mind that the increasing agricultural output often necessitates the use of agrochemicals [2, 3], which are substances used in agricultural areas and in public health programs to control pests and vectors that transmit diseases [4].

Among the most common agrochemicals, organophosphate insecticides (OPs) are considered the most toxic to vertebrates [5]. The metabolism and toxicology of these compounds in mammals have been intensively studied [6, 7]. These products are rapidly absorbed by ingestion, inhalation, percutaneous and conjunctival absorption [8, 9]. OPs are also known as neurotoxic agents, acting by covalently binding to the active site of acetylcholinesterase (AChE), an enzyme of the cholinesterase family responsible for the hydrolysis of the neurotransmitter acetylcholine (ACh) at the conclusion of the nerve impulse transmission [10].

**Fig. 1:**
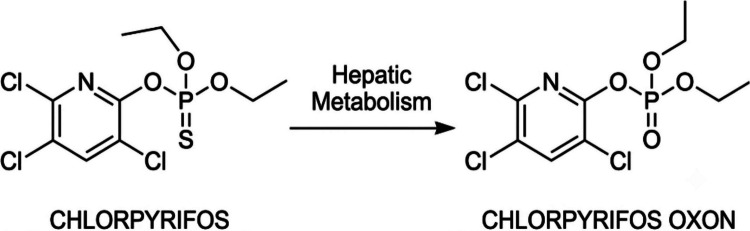
Bioactivation of chlorpyrifos to chlorpyrifos oxon [12]

OPs also have extensive agricultural applications, with several being used as anthelmintic and in the control of certain ectoparasites. Examples of these pesticides include chlorpyrifos, diazinon, dimethoate, malathion and parathion. Chlorpyrifos (CPF), phosphorothioate (O,O'-diethyl-O''-(3,5,6-trichloro-2-pyridyl)), is one of the five most commercialized OP insecticides globally, with over 900 different formulations. Once absorbed, CPF is metabolically activated to Chlorpyrifo-oxon (CPF-oxon) (Fig. 1), the most toxic form of this insecticide, which is responsible for its toxic effects in mammals [11]. After consumption of products containing chlorpyrifos residues, the original insecticide is converted into oxon in the liver or other organs, making it approximately 1,000 times more potent than the insecticide itself [12].

The absorption of OPs by the human body occurs through food consumption and also through the dermal and respiratory routes [13, 14]. When CPF is absorbed, it causes irreversible inhibition of AChE activity in the nervous system, resulting in accumulation of ACh and activation of muscarinic and nicotinic receptors, which can lead to death [13, 15]. Given its extensive use as an insecticide and its toxicity, developing effective methods for degrading it is essential [16–18].

The primary chemical transformation routes of CPF and its analogues include oxidation [19], hydrolysis [14], and nucleophilic substitution reactions [20]. A promising approach to degrade these compounds involves the use of metallic oxides. The application of metallic oxides for the degradation of OPs has been studied for several years and has proven to be a potential method for adsorbing and decontaminating these substances [21–23]. Transition metals, in particular, offer an intriguing alternative, serving as model systems that are amenable to detailed experimental and theoretical studies [24, 25].

Among the transition-metal-based materials investigated for catalytic applications, niobium oxides have attracted considerable interest owing to their electronic properties, catalytic activity, and chemical stability[21, 22]. Nb₂O₅ is one of the most extensively studied niobium oxides and has found applications in catalysis, electronics, and ceramic materials [26–30]. However, niobium dioxide (NbO₂) exhibits distinct electronic characteristics, including a significantly narrower band gap (~ 0.80 eV) than Nb₂O₅ (~ 3.4 eV), which facilitates electron transport and enhances its potential for electrochemical and catalytic applications [31–33]. In contrast, Nb₂O₅ has a band gap of around 3.4 eV, classifying it as a semiconductor with low conductivity [33]. Furthermore, the partially occupied 4 d orbital of Nb in NbO₂ contributes to its electronic conductivity and may promote interactions with reactive species during catalytic processes [33, 34].

Although metal oxides have been widely investigated for the degradation of organophosphorus compounds, the potential of NbO₂ for CPF-oxon degradation remains largely unexplored. Advances in computational chemistry have enabled detailed investigations of reaction mechanisms involving metal oxides, providing valuable insights into their reactivity and catalytic behavior. Therefore, this study employs molecular modeling methods to investigate the physicochemical parameters and possible mechanisms involved in CPF-oxon degradation mediated by NbO₂.

## Methodology

### Computational details

**Scheme 1:**
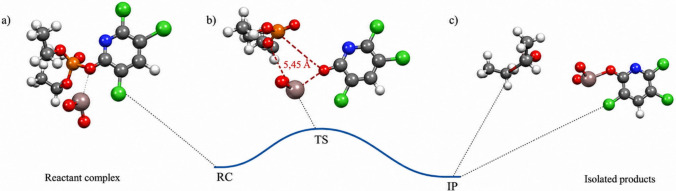
Reaction of chlorpyrifos-oxon with $Nb{O}_{2}^{2+}$. (**a**) Reactant complex, (**b**) Transition state (TS), (**c**) Isolated products. The color code for the atoms is as follows: oxygen – red, niobium – gray, nitrogen – blue, chlorine – green, carbon – dark gray, and hydrogen – white

The initial structure was built as a reactant complex composed of one NbO₂ molecule and one chlorpyrifos-oxon molecule, which was used as the starting point for subsequent geometry optimizations and transition state searches (Scheme 1).

The reactants, products, and transition state were fully optimized using density functional theory (DFT) with the functionals B3LYP [35, 36], M06-2X [37], CAM-B3LYP [38], and ωB97X-D3 [39, 40], in combination with the def2-TZVP basis set [41]. For the niobium atom, the corresponding effective core potential (ECP) def2-TZVP-ECP [41] was employed.

After preliminary single-point energy calculations with the B3LYP, M06-2X, CAM-B3LYP, and ωB97X-D3 functionals, the sensitivity of the calculated reaction profile to the level of theory was assessed. To select the most appropriate density functional for the system, single-point energy calculations were performed on the reactant, transition state, and product geometries using four functionals: B3LYP, ωB97X-D3, M06-2X, and CAM-B3LYP, in both gas and solvent phases (Tables S1a–S1c). The ωB97X-D3 functional consistently yielded the lowest absolute energies across all structures and phases, suggesting a more complete description of the electronic structure of the Nb complex. This is consistent with the known importance of dispersion corrections and range-separated exchange for systems containing second-row transition metals and mixed donor–acceptor ligands. Based on these results and its strong performance in benchmark studies of transition metal reactivity ωB97X-D3 was selected for all subsequent calculations [39, 40, 42, 43].

The NbO₂ system was represented using a molecular system with total charge + 2 and spin multiplicity 2. This approach is consistent with previous theoretical studies employing cationic niobium oxide clusters (NbOₘⁿ⁺) to investigate the intrinsic electronic structure and reactivity of niobium oxide species toward bond activation processes [44]. The present model was adopted to explore the elementary interaction between CPF-oxon and a representative niobium oxide active site rather than to reproduce the full complexity of an extended NbO₂ surface.

The initial geometry of the pre-reactive complex was constructed by placing the $Nb{O}_{2}^{2+}$ near the oxygen atom connecting the phosphoryl group to the pyridine ring (O9). This site was selected based on chemical considerations, as the positively charged niobium center can interact favorably with oxygen-containing groups, and cleavage of the P–O bond linking the phosphate moiety to the aromatic ring is a well-established degradation pathway for chlorpyrifos-derived compounds [45, 46]. This reaction pathway leads to the formation of 3,5,6-trichloro-2-pyridinol (TCP), which is widely recognized as one of the major degradation products of chlorpyrifos and chlorpyrifos-oxon and has been reported in both experimental and mechanistic studies [16, 47]. An alternative coordination mode involving the phosphoryl oxygen atom (P = O) was also investigated. However, the corresponding reaction pathway was associated with a higher activation barrier than that obtained for the O9-mediated pathway. Therefore, the pathway involving cleavage of the P–O bond connecting the phosphate group to the pyridine ring was selected for detailed mechanistic analysis. The energetic results for both pathways are provided in Table S112 of the Supplementary Information.

Geometry optimization of this arrangement led to a stable reactant complex, which was subsequently used as the starting point for the transition-state search. Although an exhaustive conformational search was not performed, the selected configuration is consistent with the proposed degradation pathway and with the electronic redistribution observed for O9 along the reaction coordinate, as discussed in the NPA analysis section.

The selected coordination mode was not intended to represent the unique or most stable adsorption geometry on NbO₂^2^⁺. Rather, it was chosen because it directly probes the P–O bond cleavage pathway leading to the formation of 3,5,6-trichloro-2-pyridinol (TCP), which is one of the major degradation products consistently reported for chlorpyrifos-derived compounds. Experimental studies have identified TCP as a primary degradation product formed through cleavage of the P–O bond linking the phosphate group to the pyridine ring, supporting the mechanistic relevance of this reaction pathway [16, 47]. Although alternative adsorption geometries may exist, the present study focuses on the mechanistic feasibility of this experimentally established degradation route. Furthermore, the assignment of the transition state was based on the analysis of the dominant imaginary frequency and the evolution of the key structural parameters associated with P–O bond cleavage, which consistently connect the pre-reactive complex to the experimentally observed degradation products.

No symmetry restrictions were imposed during the optimization process. Geometry optimizations were carried out using the “tight” convergence criteria implemented in ORCA [48], with energy changes below 10⁻^6^ Hartree and maximum gradient norms below 3 × 10⁻^4^ Hartree/Bohr. Frequency calculations were performed to confirm the nature of the stationary points. No imaginary frequencies were found for the optimized geometries of isolated reactants and products. Solvent effects were considered using the conductor-like polarizable continuum model (CPCM) [49], with water as the solvent.

For the transition state in solvent, one imaginary frequencies was identified. Frequency analysis of the transition-state structure revealed the dominant imaginary frequency associated with the P–O bond cleavage coordinate. The dominant imaginary frequency is associated with the characteristic vibrational mode of the transition state (the atomic coordinates, as well as the frequency, are provided in Supplementary Material S2–S7). Thermodynamic properties were computed through vibrational frequency analysis under standard conditions (298.15 K, 1 atm).

All calculations were carried out using ORCA [48] for geometry optimizations and frequency analyses. Natural Population Analysis (NPA) was performed with Gaussian [50] software to investigate electronic structure and charge distribution within the complex.

From the optimized geometries, the activation energy (E_a_), reaction enthalpy (ΔH), and activation free energy (Δg‡) parameters were calculated, which are essential for understanding the kinetic and thermodynamic viability of the reaction. The activation energy (E_a_), defined as the difference in energy between the transition state (E_TS_) and the reactant complex (E_R_), was calculated as shown in Eq. 1:

1 $$E_a\:=\:E_{TS}\:-\:E_R$$

The reaction enthalpy (ΔH), which reflects the difference between the enthalpy of the products (H_P_) and reactants (H_R_) complexes, was obtained as follows (Eq. 2):

2 $$\Delta H\:=\:H_P\:-\:H_R$$

The activation free energy (Δg‡) of the reaction, was calculated as the difference between the Gibbs free energy of the TS (G_TS_) and the Gibbs free energy of the reactants complex (G_R_) (Eq. 3):

3 $$\Delta g\ddagger\:=\:G_{TS}\:-\:G_R$$

Transition state (TS) theory provides a fundamental theoretical framework for estimating the reaction rate constant (k) of a chemical reaction based on the free energy of activation. The Eyring equation, derived from TS, relates the rate constant to thermodynamic parameters and temperature, enabling kinetic predictions from either computational or experimental data. The equation is expressed as:

$$k=\frac{{k}_{b}T}{h}{ . e}^{-\frac{\Delta \mathrm{G}\ddagger }{RT}}$$

Equation 4: Eyring equation for estimating the rate constant (k) of a chemical reaction.

 where

- ${k}_{b}$ the Boltzmann constant,
- h is Planck’s constant,
- T is the absolute temperature (in kelvin),
- R is the ideal gas constant,
- ΔG‡ is the activation free energy of the reaction.

In this work, the Eyring equation was used to estimate the rate constant (k) for the bond-breaking step occurring on the $Nb{O}_{2}^{2+}$

## Results

Understanding thermodynamic and kinetic parameters is crucial to elucidate the mechanisms of complex chemical reactions, such as the breakdown of chlorpyrifos-oxon using $Nb{O}_{2}^{2+}$. Such values ​​allow for a detailed analysis of reaction behavior, providing insights into the viability and efficiency of the process. These values ​​are shown in Table 1.

**Table 1:** Thermodynamic parameters of the chlorpyrifos-oxon breakdown with $NbO_2^{2+}$

Parameter	*Gas phase* Value (kcal·mol⁻^1^)	*Solvent* Value (kcal·mol⁻^1^)
Activation Energy (E_a_)	7.4	2.9
Activation free energy (Δg^*‡*^)	6.4	2.3
Reaction enthalpy (ΔH)		1.1

The activation parameters reported in this work were calculated using the optimized reactant complex as the reference state. This approach was adopted because the primary objective of the study was to investigate the elementary reaction mechanism associated with P–O bond cleavage and the role of $Nb{O}_{2}^{2+}$ in facilitating this process. Consequently, the reported energy barriers should be interpreted as relative activation parameters for the bond-breaking step occurring after formation of the reactant complex. Additional contributions associated with adsorption, diffusion, and reactant association may influence the overall kinetics of the process and are not explicitly represented within the present molecular systems. Nevertheless, the results provide valuable mechanistic insight into the electronic and energetic factors governing CPF-oxon degradation mediated by $Nb{O}_{2}^{2+}$ and establish a foundation for future studies employing more elaborate surface representations.

The activation energies were obtained from total energy calculations using DFT, considering two distinct scenarios: (i) in the gas phase, and (ii) in the presence of an implicit solvent model. The calculated ΔG‡ values were 6.4 kcal·mol⁻^1^ and 2.3 kcal·mol⁻^1^, respectively. Although the Eyring equation provides a route to estimate rate constants from activation free energy barriers its direct application to heterogeneous reactions on solid surfaces, such as bond cleavage processes on $Nb{O}_{2}^{2+}$, must be approached with caution.

While the Eyring equation provides a powerful way to connect thermodynamics and kinetics, it assumes a single, well-defined transition state and a smooth potential energy surface. These assumptions are generally valid for molecular systems such as the $Nb{O}_{2}^{2+}$–chlorpyrifos-oxon complex modeled in this study. However, they can be violated in surface-catalyzed reactions, where multiple adsorption configurations, dynamic surface rearrangements, and diffusion phenomena occur. According to Campbell (2017) [51], the apparent rate constants observed in heterogeneous catalysis often reflect the collective influence of several microscopic steps rather than a single reaction pathway. Therefore, the rate constants reported in this work should not be interpreted as direct experimental rate constants for chlorpyrifos-oxon degradation on NbO₂ surfaces. Instead, they should be regarded as apparent kinetic descriptors derived from the adopted molecular model, which are useful for evaluating the relative kinetic feasibility of the proposed reaction pathway.

The limitations of the present molecular system are acknowledged; however, previous studies have shown that, for small substrates, NbO₂^2^⁺ molecular systems mainly affect the reaction energetics rather than the reaction mechanism [52]. Therefore, the mechanism of the reaction, as well as the relative energies, can be well established with that model. The underlying assumptions of TS include a well-defined reaction coordinate and a single, dominant transition state, which often do not hold in real catalytic systems, especially those involving metal or oxide surfaces. Although these effects are expected to play an important role in real NbO₂ surfaces and heterogeneous catalytic systems, they are beyond the scope of the present molecular model.

Nevertheless, the comparison between gas-phase and solvated conditions provides valuable qualitative insight. In this study, the activation barrier was calculated as 6.4 kcal·mol⁻^1^ in the gas phase and 2.03 kcal·mol⁻^1^ with solvent. This significant reduction in energy barrier directly affects the estimated rate constant, increasing it from approximately 1.40 × 10^7^ s^−1^ to 8.57 × 10^9^ s^−1^. This significant reduction in the activation free energy from 9.09 to 6.01 kcal·mol⁻^1^ directly affects the estimated rate constant, which increases from approximately 1.40 × 10^7^ s⁻^1^ to 8.57 × 10^9^ s⁻^1^ upon inclusion of solvent effects. At this rate, the P–O bond cleavage step occurs on a timescale of approximately 10⁻^10^ s in the solvated environment, indicating a near-diffusion-limited elementary step. For context, this rate constant is comparable to those observed in enzyme-catalyzed phosphoester hydrolysis, where values in the range of 10^8^–10^10^ s⁻^1^ are reported for efficient catalytic systems. This increase of nearly three orders of magnitude highlights the strong stabilizing effect of the aqueous environment on the transition state, likely through electrostatic interactions between the polar solvent and the charged NbO₂⁺^2^ center. Therefore, the presence of NbO₂⁺^2^ in aqueous conditions significantly facilitates the degradation of CPF-oxon by lowering the activation barrier associated with the P–O bond cleavage step. Physically, the exponential dependence of *k* on ΔG‡ demonstrates how even small decreases in activation free energy can lead to dramatic rate accelerations. Chemically, this behavior suggests a stabilization of the transition state by the solvent, possibly through electrostatic interactions, reorganization of the electronic environment around the active site, or modulation of surface–reactant interactions.

However, this unusually fast reactivity may suggest that the system's reaction mechanism involves faster processes or alternative reaction pathways not fully captured in the current model. Additionally, the complexity of the NbO₂ surface, including variations in coordination, oxidation states, and structural defects, can contribute to unexpected reactivity patterns. Therefore, while the calculated trends are useful for understanding the role of solvent in enhancing reactivity, these exceptionally high-rate constants should be considered more as qualitative indicators than as accurate representations of the actual reaction rates."

For pesticide degradation processes, a low E_a_ is desirable as it allows the reaction to occur quickly and efficiently, facilitating the removal of environmental contaminants. In the case of this agrochemical’s degradation, an activation energy of approximately 8.58 without solvent and 3.4 kcal·mol⁻^1^ with solvent is considered relatively low [53]. This suggests that the reaction is energetically favorable and may proceed readily without extreme conditions. The activation energy determines the kinetic barrier that must be overcome for the reaction to proceed [54, 55].

Next, we will compare the activation energy (E_a_) and ΔH for chlorpyrifos-oxon degradation with literature values for different degradation methods and organophosphates used as agrochemicals, given the lack of experimental and theoretical data for $Nb{O}_{2}^{2+}$. This comparison is important for validating the results and situating them within the context of established findings, considering the absence of direct experimental references for niobium dioxide in the literature.

For comparison purposes, Hassan (2019) [53] conducted experiments to evaluate the sonocatalytic degradation of chlorpyrifos under different conditions. The efficiency of the degradation was investigated using composites of nanotitania and carbon nanotubes doped with zirconium cations. The authors determined the activation energy of the degradation process, with E_a_ values of 2.9 kcal·mol⁻^1^ for TiO_2_, 2.8 kcal·mol⁻^1^ for CT10, and 1.9 kcal·mol⁻^1^ for ZCT10. It should be kept in mind that niobium dioxide emerges as a versatile, cost-effective, and naturally abundant material, exhibiting activation energy values comparable to those of TiO₂, CT10 and ZCT10. These characteristics underscore its strong potential for large-scale deployment, especially in agricultural areas burdened by significant agrochemical runoff.

In this work, the reaction enthalpy for chlorpyrifos-oxon breakdown with NbO₂^2^⁺ was calculated to be slightly positive, with values of 1.7 kcal·mol⁻^1^ (gas phase) and 1.1 kcal·mol⁻^1^ (solvent phase). These small energy differences indicate a nearly thermoneutral process and fall within the typical uncertainty range associated with density functional theory calculations [42]. Therefore, the reaction cannot be conclusively classified as either strongly exothermic or strongly endothermic based solely on the calculated enthalpy values.This value is consistent with theoretical expectations and suggests that the reaction is reasonably efficient compared to the values ​​found by Aydogdu et al. (2021) [56] demonstrated that the photocatalytic degradation of organophosphorus compounds (DDMP, DEP, and ISPC) using hydroxyl radicals and TiO₂ under UV irradiation was highly effective in both gas phase and aqueous media. Theoretical calculations revealed exothermic reactions, with enthalpy values ranging from −18.34 kcal·mol⁻^1^ (DDMP) to −16.20 kcal·mol⁻^1^ (DEP) in the gas phase, and from −17.00 kcal·mol⁻^1^ (DDMP) to −17.30 kcal·mol⁻^1^ (ISPC) in aqueous solution. Experimentally, the method proved remarkably efficient, achieving degradation rates of 83–90% within just 90–100 min of treatment, confirming its effectiveness for pollutant removal across different media.

Similarly, the degradation of chlorpyrifos-oxon by NbO₂^2^⁺ was found to exhibit only a small enthalpic change, suggesting that the process remains thermodynamically accessible despite its slightly endothermic character. Small reaction enthalpies of this magnitude are generally considered insufficient to alter the qualitative thermodynamic interpretation of a reaction pathway, particularly when accompanied by favorable Gibbs free energy profiles and low activation barriers [42, 55]. Consequently, the viability of the proposed degradation mechanism is primarily supported by its favorable kinetic characteristics rather than by a large enthalpic driving force. Understanding these energetic contributions remains important for assessing reaction feasibility, intermediate stability, and the overall behavior of the degradation process.

The results obtained in this work indicate that $Nb{O}_{2}^{2+}$ has significant potential for the degradation of chlorpyrifos-oxon, compared to existing literature [14, 56, 57]. Using $Nb{O}_{2}^{2+}$ for pesticide breakdown makes good use of niobium, which is abundant, and can help improve waste treatment and pollution control. This can be helpful for agriculture and industries aimed at sustainability and saving energy. Additional experimental investigations into thermodynamic and kinetic parameters will support the development of safer and more effective treatment strategies, contributing to food security and public health in the face of increasing agricultural and population demands.

## Reaction Path

An experimental study carried out by Bootharaju and Pradeep [45] aimed to understand in detail the mechanism of interaction between nanoparticles (NPs) of noble metals, such as silver (Ag) and gold (Au), and the organophosphorothioate pesticide chlorpyrifos. They observed that CPF decomposes in the presence of Ag and Au NPs, both in supported and unsupported forms. This observation suggests a chemical interaction between the NPs and the pesticide that leads to its degradation. To understand this mechanism, the authors employed several analytical techniques. The analysis revealed that CPF degradation occurs through the formation of a surface complex between the Ag NPs and the sulfur atom (S) present in the pesticide molecule. This complex was confirmed by Raman spectroscopy, which showed a new peak at 248 cm^−1^ attributed to the Ag–S interaction. Within this surface complex, the P-O bond in the CPF molecule breaks, leading to the formation of two degradation products: 3,5,6-trichloro-2-pyridol (TCP), a stable aromatic species, and diethyl thiophostat (DETP). This degradation route was identified by ES-MS analysis [45].

**Scheme 2:**
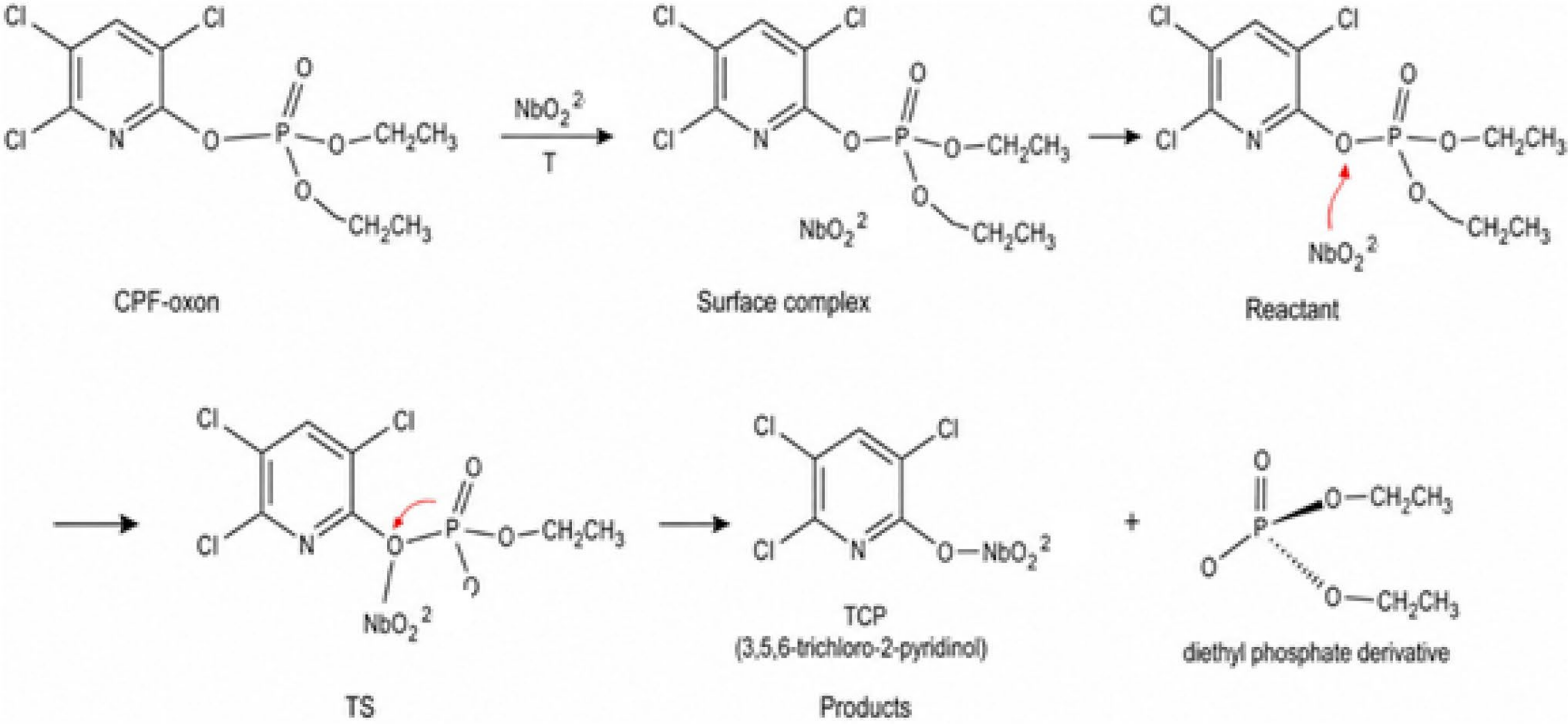
Representation of degradation of CPF-oxon on $Nb{O}_{2}^{2+}$ (adapted from Bootharaju [45]). The red arrows indicate the proposed redistribution of electron density associated with Nb–O bond formation and P–O bond cleavage and do not represent a formal electron-transfer process

Based on these experimental results considering chlorpyrifos-oxon, the most toxic form of this agrochemical, the reaction pathway proposed in Scheme 2 can be rationalized as follows. Initially, chlorpyrifos-oxon (CPF-oxon) interacts with the $Nb{O}_{2}^{2+}$ molecular systems at room temperature. In this step, the oxygen atom connecting the phosphate group to the pyridine ring coordinates to the niobium center. This oxygen atom is directly involved in the P–O bond cleavage that leads to the formation of the degradation products and therefore represents a chemically relevant interaction site. The presence of lone electron pairs on oxygen favors coordination with the electrophilic niobium center, leading to formation of the initial reactant complex. The positively charged nature of the Nb center throughout the reaction coordinate, as indicated by the NPA analysis, further supports this interaction. Consequently, in the transition state, formation of the Nb–O interaction is accompanied by elongation and subsequent cleavage of the P–O bond of the phosphoric ester, initiating the degradation process. From a mechanistic perspective, the reaction is characterized by $Nb{O}_{2}^{2+}$-assisted P–O bond cleavage and can be viewed as a substitution-type degradation pathway rather than a direct oxidation process.

**Fig. 2:**
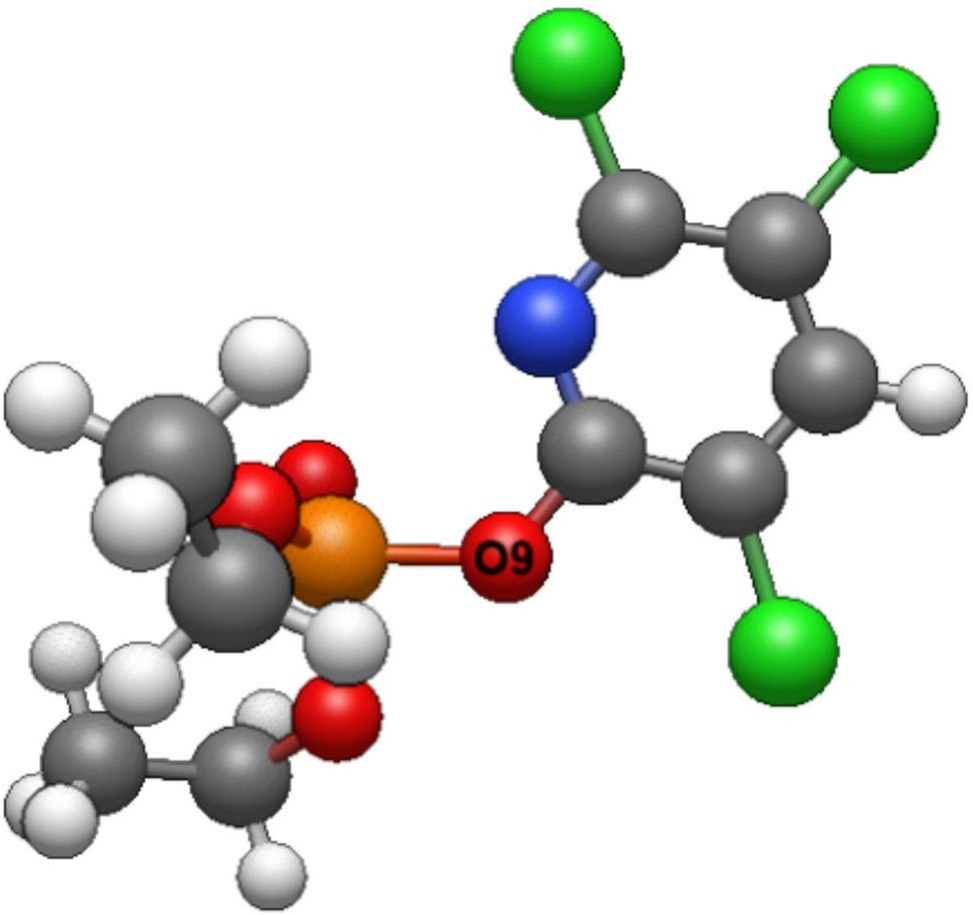
Position of oxygen atom O9 in the CPF-oxon structure, which is conserved in the CPF molecule as well

The NPA (Table 2, 3 and S9-S11) data reveal distinct electronic dynamics between solvent and gas-phase systems. In the gas phase, we observe more abrupt changes in charge distribution between Nb and O9 during P-O bond cleavage, with niobium showing more pronounced charge variations (+ 0.8275 e → + 0.9002 e → + 0.8216 e) compared to the solvated system. This suggests the solvent acts as an electronic moderator, dampening the extreme charge fluctuations characteristic of the gas-phase reaction. These electronic shifts are closely associated with the behavior of oxygen atom O9, whose position is illustrated in Fig. 2. Notably, this atom occupies an equivalent position in both CPF-oxon and CPF structures.

**Table 2:** Summary of Natural Population Analysis for Niobium and Oxygen 9 Atom in reactant, TS and product in solvent phase, highlighting key electronic properties and charges

Property	Reactant		TS		Product	
	O	Nb	O	Nb	O	Nb
Natural Charge (e)	−0.33	0.81	−0.41	0.82	−0.28	0.94
Valence Electrons	3.39	1.68	3.39	1.68	3.26	1.57
Rydberg Contribution	0.012	0.016	0.01	0.02	0.013	0.004

**Table 3:** Summary of Natural Population Analysis for Niobium and Oxygen 9 Atom in reactant, TS and product in gas phase, highlighting key electronic properties and charges

Property	Reactant		TS		Product	
	O	Nb	O	Nb	O	Nb
Natural Charge (e)	−0.331	0.81	−0.23	0.90	−0.30	0.82
Valence Electrons	3.320	1.70	3.22	1.62	3.29	1.69
Rydberg Contribution	0.012	0.016	0.008	0.011	0.012	0.017

The nucleophilic oxygen (O9) exhibits markedly different behavior in the two environments. While its charge shifts significantly in the gas phase (−0.4114 e → −0.2358 e in TS), the solvated system shows a more gradual transition (−0.3314 e → −0.4114 e). This solvent-mediated moderation appears to facilitate a smoother charge redistribution process, potentially correlating with the experimentally observed lower activation energy in solvated conditions.

Valence electron populations show significant environmental dependence. The gas-phase Nb experiences a steeper decline in valence electrons (1.6865 e → 1.6220 e) versus the solvated system (1.70017 e → 1.68655 e), indicating enhanced electronic delocalization in the absence of solvent. Conversely, gas-phase O9 loses more electron density (3.3998 e → 3.2275 e) than its solvated counterpart (3.31962 e → 3.39983 e), demonstrating how solvent helps preserve this reactive center's nucleophilic character.

Rydberg contributions reveal particularly interesting trends. The gas-phase system shows sharper reduction in Nb's Rydberg contribution during TS (0.0110 e → 0.0084 e), while the solvated system maintains greater delocalization (0.0162 e → 0.0110 e). This implies solvent molecules help stabilize charge distribution even at critical reaction stages, possibly through intermediate stabilization.

The final product states differ substantially between systems. The gas-phase yields a more covalent Nb–O interaction (Nb + 0.8216 e, O9 −0.3073 e) compared to the solvated product (Nb + 0.94866 e, O9 −0.28099 e). This charge distribution difference may significantly impact subsequent product stability, with gas-phase conditions generating potentially more reactive species.

This comparative analysis suggests that while both systems share the fundamental Nb → O9 charge redistribution mechanism for P-O bond activation, the solvent strongly modulates this process. The gas-phase favors more extreme, rapid charge redistribution, while the solvated environment provides better-controlled reaction pathways, moderating electronic fluctuations and better preserving O9's nucleophilic character throughout the reaction coordinate. These findings provide valuable insights for designing solvent-specific catalytic systems tailored to desired reaction outcomes.

The difference in electronegativity and charge distribution between these atoms creates a strong dipole which facilitates orbital overlap and stabilizes the transition state. This polarization effect likely lowers the activation barrier for the cleavage of the P–O bond of the phosphoric ester, enhancing the reaction efficiency. The observed charge redistribution confirms that niobium not only acts as an electrophilic center but also influences the charge redistribution within the system, making it a critical factor in the reaction mechanism. These results should be interpreted as evidence of electronic redistribution along the reaction coordinate rather than a formal electron-transfer process. The NPA analysis reveals variations in the charge distribution of Nb and O9 during the reaction, supporting the proposed bond activation and cleavage mechanism illustrated in Scheme 2.

A study conducted by Truong et al.[46] aimed to investigate the degradation mechanisms of chlorpyrifos when exposed to the hydroxyl radical (OH•) in both gas and aqueous phases. For the purposes of comparison, we considered their results obtained under aqueous-phase conditions, which align with the conditions used in our study. The methodology involved kinetic analysis of the reactions of OH^.^ addition to CPF, using Gibbs free energy and activation energy calculations. The methodology involved a kinetic analysis of the reactions between OH• radicals and CPF, carried out through calculations of Gibbs free energy and activation energy. When comparing the ΔG‡ values obtained in this study (7.4 and 2.9 kcal·mol⁻^1^) with those reported in previous works by the authors [49], it is observed that these values are close to the average range reported for the addition of OH• to the carbon atom in the pyridine ring of CPF, which lies between 8.12 and 9.75 kcal·mol⁻^1^. They identified one of the products as 3,5,6-trichloro-2-pyridinol, indicating that cleavage of the P–O bond linking the phosphate group to the aromatic ring occurs, consistent with the reaction pathway proposed for the degradation of CPF-oxon on NbO₂^2^⁺ in the present work.

The slightly higher value found in this study, compared to the lower value reported by Truong et al. [46] (8.12 kcal·mol⁻^1^), can be explained by the fact that CPF-oxon, in which sulfur is replaced by an oxygen atom is being evaluated. The increased electronegativity of oxygen compared to sulfur can lead to a redistribution of charges and changes in electronic tension, which raise the activation energy.

NPA reveals that oxygen in CPF-oxon has a charge of −1.0683 e, while sulfur in CPF carries a charge of −0.58429 e, as can be seen in Table 4 and Table S9a-b in Supplementary Information. Similar to the Mulliken analysis, NPA demonstrates that the higher electronegativity of oxygen relative to sulfur leads to a greater concentration of electron density around oxygen in CPF-oxon, influencing the reactivity of both molecules.

**Table 4:** Comparison of NPA charges of Oxygen in CPF-oxon and Sulfur in CPF

Atom	Molecule	Charge (e)
O	CPF-oxon	−1.0683
S	CPF	−0.5842

From Mulliken charge analysis, which highlighted a more negative charge on the oxygen atoms in CPF-oxon compared to the sulfur atom in CPF, indicating an intramolecular redistribution of electron density, the NPA further supports this electronic differentiation. As presented in Table 3 and Table S6 in the Supplementary Information, the oxygen atom in CPF-oxon exhibits a charge of –1.0683 e, while the sulfur atom in CPF shows a comparatively less negative charge of –0.5842 e. This contrast underscores the effect of oxygen’s higher electronegativity relative to sulfur, promoting stronger electron localization in CPF-oxon. The resulting increase in electron density around the phosphoryl group may enhance its electrophilic character, potentially influencing the compound’s interaction with biological nucleophiles. Taken together, these charge distribution patterns provide an understanding of the distinct reactivity profiles and toxicological behavior of CPF and CPF-oxon.

Moreover, the activation energy for this reaction is relatively low, in gas phase and in water, respectively, indicating that the reaction can proceed readily under ambient conditions. This low activation energy implies that the energy barrier for the reaction is easily overcome, further supporting the efficiency and feasibility of the degradation process.

In this context, our findings highlight that niobium dioxide—a versatile, inexpensive, and naturally abundant material—shows great promise for enabling the efficient degradation of chlorpyrifos-oxon. Compared to silver nanoparticles [45], niobium dioxide presents a more cost-effective alternative with superior thermodynamic properties. Its natural abundance and economic viability, combined with robust performance in facilitating chlorpyrifos-oxon degradation, positions it as a sustainable solution for large-scale environmental remediation. Considering niobium dioxide's catalytic potential, the material may promote efficient bond cleavage through surface-mediated redox cycles while maintaining structural stability. This is supported by consistent thermodynamic and kinetic parameters that validate the proposed degradation mechanism. The observed product stability and favorable energy profiles further sugest $Nb{O}_{2}^{2+}$ effectiveness in chlorpyrifos-oxon breakdown, highlighting its promise as an advanced material for environmental applications that could reduce dependence on noble metal catalysts like silver.

## Conclusion

The calculated thermodynamic and kinetic parameters demonstrate that the degradation of chlorpyrifos-oxon mediated by $Nb{O}_{2}^{2+}$ is energetically favorable and proceeds with a low activation barrier. These findings reinforce the catalytic efficiency of $Nb{O}_{2}^{2+}$ and its potential as a cost-effective material for environmental remediation. Beyond its favorable energetics, the natural abundance and low cost of niobium make NbO₂ particularly attractive for large-scale applications. In practical terms, this material could be explored in wastewater treatment systems and soil remediation strategies aimed at removing persistent agrochemical residues. Future studies combining computational and experimental approaches could refine the understanding of the degradation pathways and support the development of optimized ${\mathrm{NbO}}_{2}^{2+}$-based technologies for sustainable pollution control. Such advances would contribute directly to environmental protection, agricultural safety, and public health.

## Supplementary Information

Below is the link to the electronic supplementary material.

ESM 1 (DOCX 545 KB)

## Data Availability

The data supporting the findings of this study are available in the article and its Supplementary Information.
