# Supplementary material for: Agrochemical residues and food safety: In-depth in silico assessment of the degradation mechanism of chlorpyrifos-oxon by niobium dioxide (NbO₂), a naturally occurring and cost-effective mineral
Source: J Mol Model. 2026 Jul 31;32(8):290. doi: 10.1007/s00894-026-06865-7 (PMC13428007; doi:10.1007/s00894-026-06865-7)
Supplement: Supplementary file 1 — (DOCX 545 KB) [file 894_2026_6865_MOESM1_ESM.docx]

**Supplementary Information**

**Agrochemical residues and food safety: In-depth in silico assessment of the degradation mechanism of chlorpyrifos-oxon by niobium dioxide (NbO₂), a naturally occurring and cost-effective mineral**

Letícia S. Braga.^1^ Adelia J. A. Aquino^2*^. Teodorico C. Ramalho^1*^

^1^ Department of Chemistry. Universidade Federal de Lavras. Campus Universitário. Lavras - MG. 37200-000. Brazil

^2^ Department of Mechanical Engineering. Texas Tech University. Lubbock. TX 79409-1021 USA.

^3^ Department of Chemistry, Faculty of Science, University of Hradec Kralove, Hradec Kralove, Czech Republic.

*Corresponding authors: [adelia.aquino@ttu.edu](mailto:adelia.aquino@ttu.edu). [teo@ufla.br](mailto:teo@ufla.br)

**Table 1a.** Energetic Values of Reactants in Gas Phase and Solvent Phase

| Reactant (Gas Phase) | Energy (Ha) |  | Reactant (solvent) | Energy (Hatree) |
| --- | --- | --- | --- | --- |
| B3LYP[1] | -2.633.8921 |  | B3LYP[1] | -2.634.1632 |
| ωB97X-D3[2, 3] | -2.634.2871 |  | ωB97X-D3[2, 3] | -2.634.4910 |
| M062x[4] | -2.634.0464 |  | M062x[4] | -2.634.3172 |
| CAM-B3LYP [5] | -2.634.1997 |  | CAM-B3LYP [5] | -2.634.4731 |

**Table 1b.** Energetic Values of TS in Gas Phase and Solvent Phase

| TS (Gas Phase) | Energy (Ha) |  | TS (solvent) | Energy (Ha) |
| --- | --- | --- | --- | --- |
| B3LYP[1] | -2.633.8873 |  | B3LYP[1] | -2.633.8873 |
| ωB97X-D3[2, 3] | -2.634.2753 |  | ωB97X-D3[2, 3] | -2634,4957 |
| M062x[4] | -2.634.0200 |  | M062x[4] | -2.634.0200 |
| CAM-B3LYP [5] | -2.634.1865 |  | CAM-B3LYP [5] | -2.634.1865 |

**Table 1c.** Energetic Values of Products in Gas Phase and Solvent Phase

| Product (Gas phase) | Energy (Ha) |  | Product (solvent) | Energy (Ha) |
| --- | --- | --- | --- | --- |
| B3LYP[1] | -2.633.8993 |  | B3LYP[1] | -2.634.1385 |
| ωB97X-D3[2, 3] | -2.634.4029 |  | ωB97X-D3[2, 3] | -2.634.6425 |
| M062x[4] | -2.634.0288 |  | M062x[4] | -2.634.2688 |
| CAM-B3LYP [5] | -2.634.3161 |  | CAM-B3LYP [5] | -2.634.5570 |

**Table SI2a.** Vibrational frequency spectrum of the Nb reagent (gas phase).

| **Frequency (cm^-1^)** |
| --- |
| 656.66 |

**Table SI2b.** Vibrational frequency spectrum of the CPF-oxon reagent (gas phase).

| **Frequency (cm^-1^)** |
| --- |
| 20.95 |


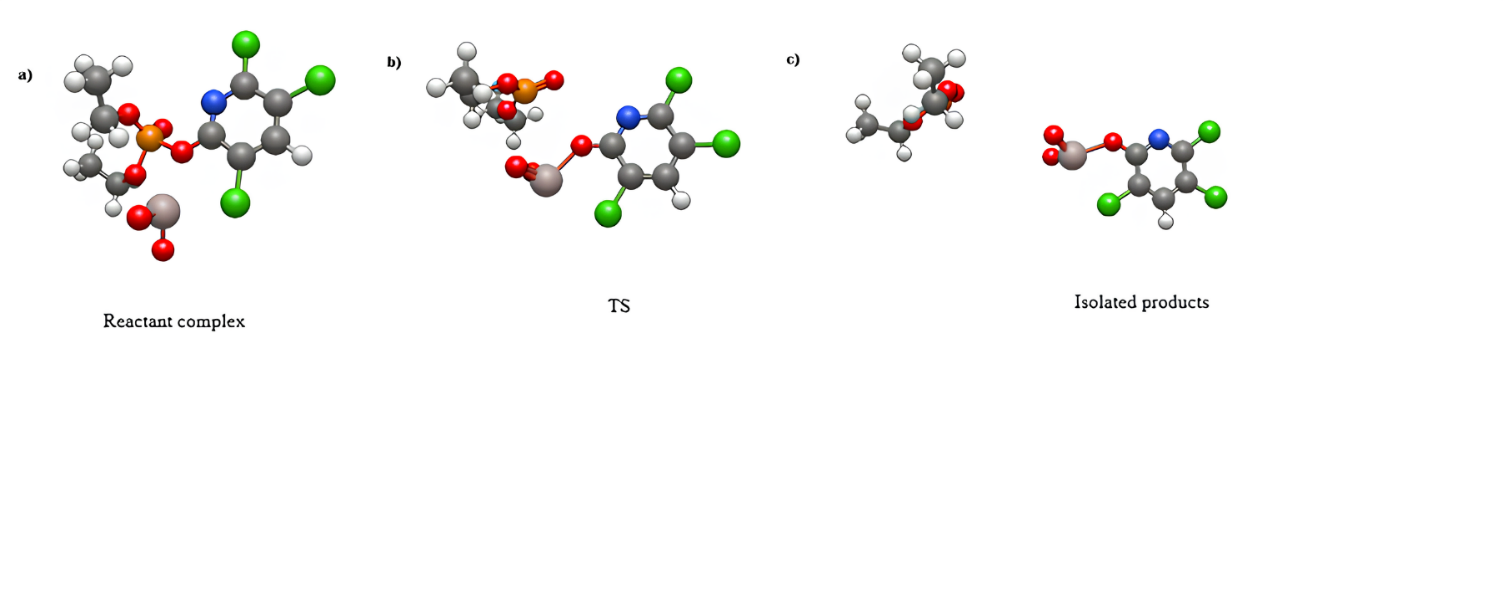


Figure S1: isolated reagents.

**Table SI2c:** Coordinates of the reagent complex (gas phase).

| P | -1.09299 | 1.39416 | -0.45936 |
| --- | --- | --- | --- |
| O | -2.42198 | 0.47811 | -0.66063 |
| O | -1.10482 | 2.04644 | 0.91385 |
| C | -1.68567 | 1.59260 | 2.22650 |
| C | -1.00800 | 2.37721 | 3.31215 |
| C | -3.57822 | 1.05588 | -1.54504 |
| C | -4.15243 | 2.28132 | -0.90787 |
| O | -0.62734 | 2.10858 | -1.62237 |
| O | -0.19308 | -0.08683 | -0.22104 |
| C | 1.61900 | -1.57406 | -0.33440 |
| C | 2.97011 | -1.78296 | -0.32711 |
| C | 3.81598 | -0.66666 | -0.17892 |
| C | 3.20746 | 0.64783 | -0.03026 |
| C | 1.12311 | -0.23481 | -0.19087 |
| N | 1.89608 | 0.80490 | -0.03837 |
| Cl | 4.16008 | 2.00655 | 0.16341 |
| Cl | 5.47133 | -0.86614 | -0.17472 |
| Cl | 0.49977 | -2.87899 | -0.53575 |
| Nb | -2.01776 | -1.68835 | -0.30512 |
| O | -2.99630 | -2.64369 | -1.31984 |
| O | -2.45182 | -2.04853 | 1.30290 |
| H | -1.51528 | 0.51923 | 2.30498 |
| H | -2.75161 | 1.79334 | 2.15568 |
| H | -1.43964 | 2.07949 | 4.27015 |
| H | 0.06259 | 2.17508 | 3.34337 |
| H | -1.16764 | 3.44714 | 3.18394 |
| H | -3.10742 | 1.23013 | -2.50876 |
| H | -4.26325 | 0.21457 | -1.58914 |
| H | -4.97110 | 2.62162 | -1.54843 |
| H | -4.56786 | 2.07516 | 0.07792 |
| H | -3.43216 | 3.09989 | -0.85598 |
| H | 3.39744 | -2.77156 | -0.43828 |

**Table SI3a:** Vibrational frequency spectrum of the transition state (gas phase).

| **Frequency (cm^-1^)** | **Intensity (KM/mol)** |
| --- | --- |
| -23.69 | 0.00 |


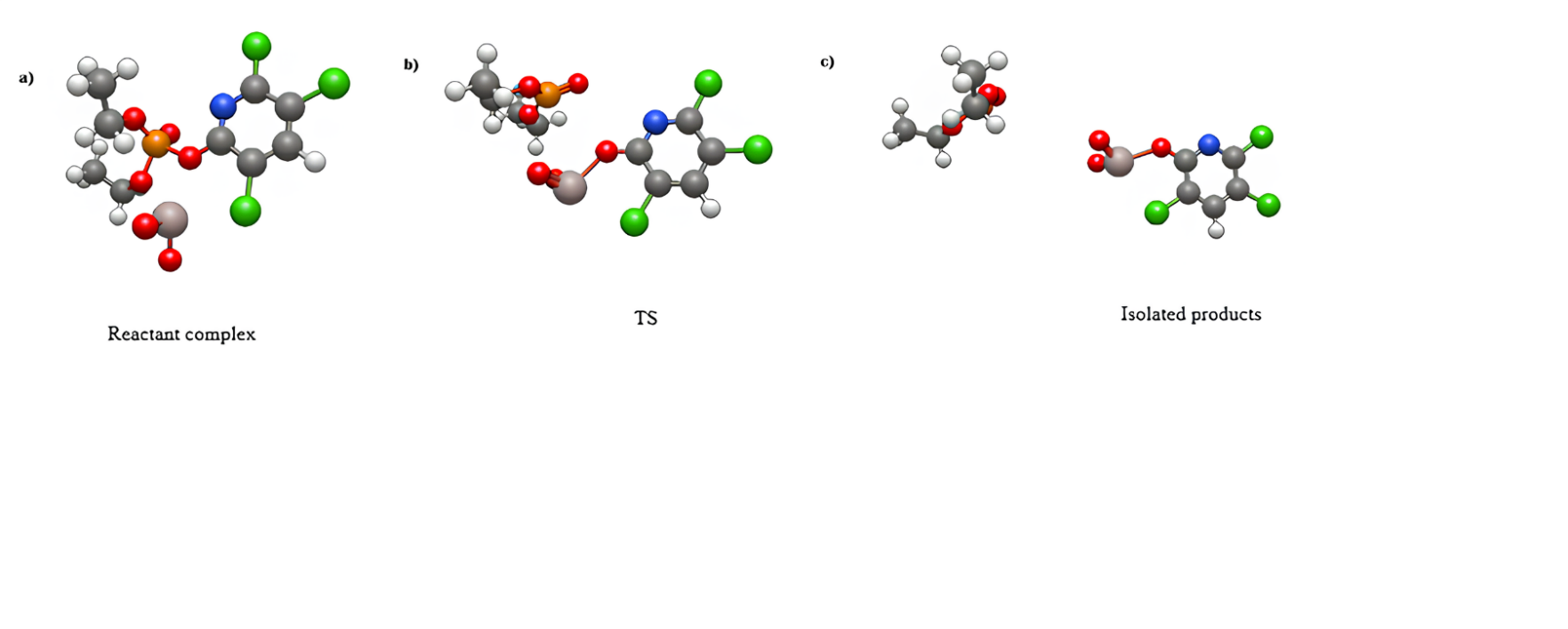


Figure S2: transition state.

**Table SI3b:** Coordinates of the transition state (gas phase).

| P | -0.90080 | 1.57695 | -0.20900 |
| --- | --- | --- | --- |
| O | -2.25410 | 1.08633 | -0.64790 |
| O | -0.81478 | 1.70287 | 1.29766 |
| C | -1.87993 | 1.44598 | 2.37609 |
| C | -1.20045 | 1.61375 | 3.69970 |
| C | -2.75871 | 0.80937 | -2.07609 |
| C | -3.90836 | 1.72879 | -2.35711 |
| O | 0.21759 | 1.88335 | -1.06810 |
| O | 1.32420 | -1.37891 | -0.20106 |
| C | 3.02324 | -2.98856 | -0.58574 |
| C | 4.31102 | -3.25787 | -0.94495 |
| C | 5.13179 | -2.17441 | -1.31262 |
| C | 4.54855 | -0.83786 | -1.29504 |
| C | 2.51832 | -1.62895 | -0.57367 |
| N | 3.31285 | -0.61963 | -0.94259 |
| Cl | 5.49555 | 0.48696 | -1.74710 |
| Cl | 6.72452 | -2.44292 | -1.75330 |
| Cl | 1.93678 | -4.27386 | -0.11307 |
| Nb | -0.28750 | -2.51670 | 0.40879 |
| O | -1.49642 | -1.97685 | -0.66287 |
| O | -0.67154 | -1.82246 | 1.92598 |
| H | -2.23608 | 0.43639 | 2.18981 |
| H | -2.65524 | 2.18319 | 2.18391 |
| H | -1.95002 | 1.44793 | 4.47803 |
| H | -0.40494 | 0.88182 | 3.83433 |
| H | -0.80295 | 2.62051 | 3.82285 |
| H | -1.90319 | 0.95320 | -2.73165 |
| H | -3.02589 | -0.24030 | -2.00925 |
| H | -4.32569 | 1.44388 | -3.32672 |
| H | -4.69618 | 1.62473 | -1.61176 |
| H | -3.59475 | 2.77061 | -2.42318 |
| H | 4.70972 | -4.26412 | -0.95142 |

**Table SI4a.** Vibrational frequency spectrum for product 2 (gas phase).

| **Frequency (cm^-1^)** |
| --- |
| 18.7 |

**Table SI4b:** Vibrational frequency spectrum for product 1 (gas phase).

| **Frequency (cm^-1^)** |
| --- |
| 30.21 |


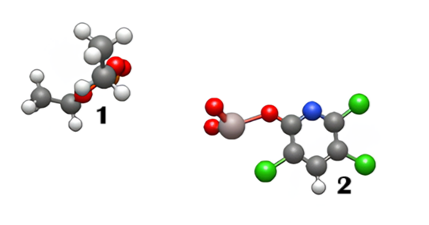


Figure S3: Isolated products 1 and 2.

**Table SI4c.** Coordinates of the products complex (gas phase).

|  |  |  |  |
| --- | --- | --- | --- |
| P | -1.90393 | 2.53069 | -0.49717 |
| O | -3.24893 | 1.64606 | -0.64154 |
| O | -1.91710 | 3.28763 | 0.81103 |
| C | -2.59594 | 3.05573 | 2.11689 |
| C | -1.57254 | 3.19608 | 3.20594 |
| C | -4.35672 | 2.15624 | -1.59085 |
| C | -4.96406 | 3.40925 | -1.04458 |
| O | -1.39080 | 3.13068 | -1.69652 |
| O | -0.64623 | 0.50582 | -0.30060 |
| C | 1.11770 | -1.02893 | -0.17691 |
| C | 2.45419 | -1.26685 | -0.10230 |
| C | 3.32742 | -0.15868 | -0.04381 |
| C | 2.75099 | 1.17977 | -0.05397 |
| C | 0.65684 | 0.33366 | -0.19458 |
| N | 1.44626 | 1.36512 | -0.12232 |
| Cl | 3.72683 | 2.51637 | 0.02563 |
| Cl | 4.96181 | -0.39032 | 0.03508 |
| Cl | -0.02823 | -2.30782 | -0.28098 |
| Nb | -2.54512 | -0.40340 | -0.30274 |
| O | -3.52659 | -1.38853 | -1.25822 |
| O | -3.04460 | -0.55815 | 1.30292 |
| H | -3.06316 | 2.07051 | 2.09171 |
| H | -3.36213 | 3.82573 | 2.16042 |
| H | -2.07572 | 3.10176 | 4.16913 |
| H | -0.81237 | 2.41583 | 3.14654 |
| H | -1.09358 | 4.17335 | 3.16604 |
| H | -3.85630 | 2.28734 | -2.54818 |
| H | -5.03999 | 1.31101 | -1.62672 |
| H | -5.76222 | 3.71108 | -1.72613 |
| H | -5.40563 | 3.25483 | -0.06063 |
| H | -4.24731 | 4.23293 | -1.01507 |
| H | 2.86018 | -2.27184 | -0.09579 |

**Table SI5a.** Vibrational frequency spectrum of the Nb reagent (solvent).

| **Frequency (cm^-1^)** |
| --- |
| 890.86 |

**Table SI5b.** Vibrational frequency spectrum of the CPF-oxon reagent (solvent).

| **Frequency (cm^-1^)** |
| --- |
| 12.43 |


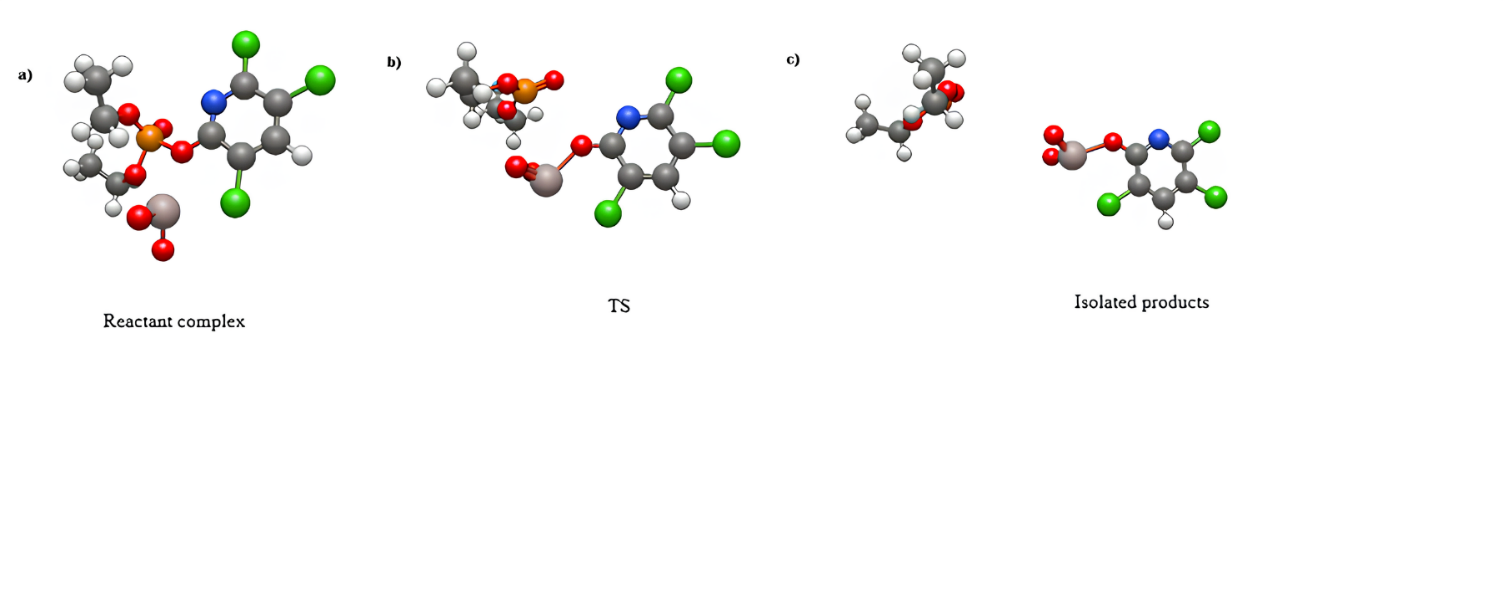


Figure S1: isolated reagents.

**Table SI5c:** Coordinates of the reagent complex (solvent).

| P | -1.09299 | 1.39416 | -0.45936 |
| --- | --- | --- | --- |
| O | -2.42198 | 0.47811 | -0.66063 |
| O | -1.10482 | 2.04644 | 0.91385 |
| C | -1.68567 | 1.59260 | 2.22650 |
| C | -1.00800 | 2.37721 | 3.31215 |
| C | -3.57822 | 1.05588 | -1.54504 |
| C | -4.15243 | 2.28132 | -0.90787 |
| O | -0.62734 | 2.10858 | -1.62237 |
| O | -0.19308 | -0.08683 | -0.22104 |
| C | 1.61900 | -1.57406 | -0.33440 |
| C | 2.97011 | -1.78296 | -0.32711 |
| C | 3.81598 | -0.66666 | -0.17892 |
| C | 3.20746 | 0.64783 | -0.03026 |
| C | 1.12311 | -0.23481 | -0.19087 |
| N | 1.89608 | 0.80490 | -0.03837 |
| Cl | 4.16008 | 2.00655 | 0.16341 |
| Cl | 5.47133 | -0.86614 | -0.17472 |
| Cl | 0.49977 | -2.87899 | -0.53575 |
| Nb | -2.01776 | -1.68835 | -0.30512 |
| O | -2.99630 | -2.64369 | -1.31984 |
| O | -2.45182 | -2.04853 | 1.30290 |
| H | -1.51528 | 0.51923 | 2.30498 |
| H | -2.75161 | 1.79334 | 2.15568 |
| H | -1.43964 | 2.07949 | 4.27015 |
| H | 0.06259 | 2.17508 | 3.34337 |
| H | -1.16764 | 3.44714 | 3.18394 |
| H | -3.10742 | 1.23013 | -2.50876 |
| H | -4.26325 | 0.21457 | -1.58914 |
| H | -4.97110 | 2.62162 | -1.54843 |
| H | -4.56786 | 2.07516 | 0.07792 |
| H | -3.43216 | 3.09989 | -0.85598 |
| H | 3.39744 | -2.77156 | -0.43828 |

**Table SI6a:** Vibrational frequency spectrum of the transition state (solvent).

| **Frequency (cm^-1^)** |
| --- |
| -18.14 |


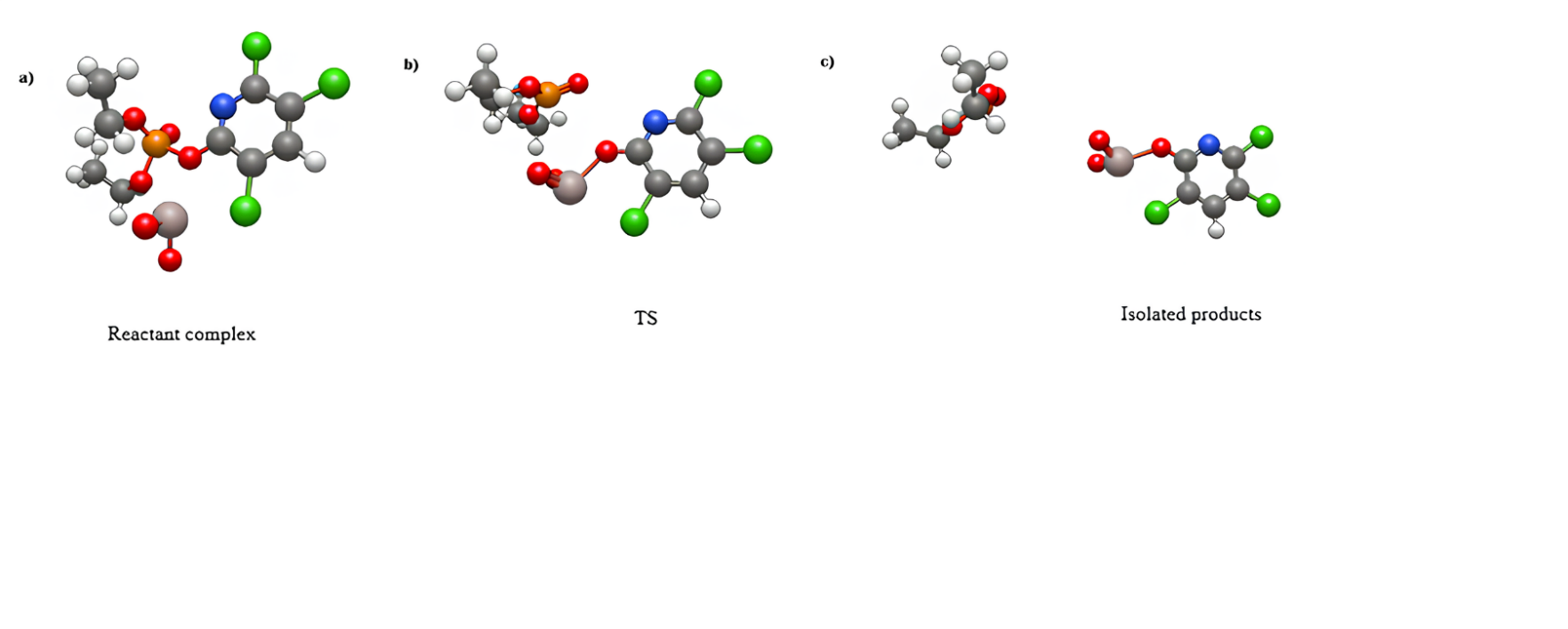


Figure S2: transition state.

**Table SI6b:** Coordinates of the transition state (solvent).

| P | -5.31264 | 0.14248 | -0.16226 |
| --- | --- | --- | --- |
| O | -6.10753 | -1.10501 | 0.18348 |
| O | -5.89664 | 0.85722 | -1.35556 |
| C | -7.15913 | 0.55209 | -2.18047 |
| C | -8.27434 | 1.42326 | -1.68913 |
| C | -5.83479 | -2.06227 | 1.35680 |
| C | -6.83388 | -1.78498 | 2.43869 |
| O | -4.14073 | 0.60526 | 0.54219 |
| O | 2.04106 | 0.69861 | 0.42950 |
| C | 3.37619 | -0.99735 | -0.57666 |
| C | 4.65233 | -1.42468 | -0.87338 |
| C | 5.73926 | -0.64369 | -0.48895 |
| C | 5.46977 | 0.60029 | 0.21563 |
| C | 3.20435 | 0.26274 | 0.13281 |
| N | 4.25833 | 0.99758 | 0.49281 |
| Cl | 6.75659 | 1.58729 | 0.70516 |
| Cl | 7.30941 | -1.14462 | -0.84009 |
| Cl | 2.01343 | -1.90670 | -1.01986 |
| Nb | 0.02982 | 0.88016 | 0.64079 |
| O | -0.61279 | -0.47607 | 1.46772 |
| O | -0.74351 | 0.85078 | -0.88840 |
| H | -7.34267 | -0.51416 | -2.07501 |
| H | -6.81780 | 0.79029 | -3.18343 |
| H | -9.13875 | 1.24820 | -2.33497 |
| H | -8.56682 | 1.17328 | -0.66857 |
| H | -8.01386 | 2.47883 | -1.75249 |
| H | -4.80055 | -1.89884 | 1.65134 |
| H | -5.95358 | -3.03070 | 0.87971 |
| H | -6.67219 | -2.51655 | 3.23443 |
| H | -6.70069 | -0.79167 | 2.86867 |
| H | -7.85558 | -1.89996 | 2.07901 |
| H | 4.81342 | -2.35576 | -1.39893 |

**Table SI7a.** Vibrational frequency spectrum for product 1 (solvent).

| **Frequency (cm^-1^)** |
| --- |
| 33.09 |

**Table SI7b:** Vibrational frequency spectrum for product 2 (solvent).

| **Frequency (cm^-1^)** |
| --- |
| 12.44 |


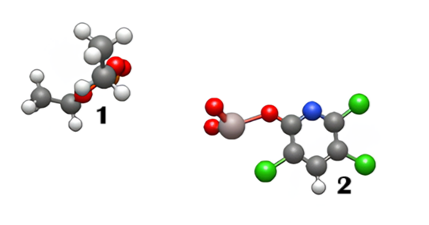


Figure S3: Isolated products 1 and 2.

**Table SI7c.** Coordinates of the products complex (solvent).

| P | -0.71538 | 1.53044 | -0.31906 |
| --- | --- | --- | --- |
| O | -2.06868 | 1.03982 | -0.75796 |
| O | -0.62936 | 1.65636 | 1.18760 |
| C | -1.69451 | 1.39947 | 2.26603 |
| C | -1.01503 | 1.56724 | 3.58964 |
| C | -2.57329 | 0.76286 | -2.18615 |
| C | -3.72294 | 1.68228 | -2.46717 |
| O | 0.40301 | 1.83684 | -1.17816 |
| O | 1.32420 | -1.37891 | -0.20106 |
| C | 3.02324 | -2.98856 | -0.58574 |
| C | 4.31102 | -3.25787 | -0.94495 |
| C | 5.13179 | -2.17441 | -1.31262 |
| C | 4.54855 | -0.83786 | -1.29504 |
| C | 2.51832 | -1.62895 | -0.57367 |
| N | 3.31285 | -0.61963 | -0.94259 |
| Cl | 5.49555 | 0.48696 | -1.74710 |
| Cl | 6.72452 | -2.44292 | -1.75330 |
| Cl | 1.93678 | -4.27386 | -0.11307 |
| Nb | -0.41657 | -1.98781 | 0.38857 |
| O | -1.62549 | -1.44796 | -0.68309 |
| O | -0.80061 | -1.29357 | 1.90576 |
| H | -2.05066 | 0.38988 | 2.07975 |
| H | -2.46982 | 2.13668 | 2.07385 |
| H | -1.76460 | 1.40142 | 4.36797 |
| H | -0.21952 | 0.83531 | 3.72427 |
| H | -0.61753 | 2.57400 | 3.71279 |
| H | -1.71777 | 0.90669 | -2.84171 |
| H | -2.84047 | -0.28681 | -2.11931 |
| H | -4.14027 | 1.39737 | -3.43678 |
| H | -4.51076 | 1.57822 | -1.72182 |
| H | -3.40933 | 2.72410 | -2.53324 |
| H | 4.70972 | -4.26412 | -0.95142 |

**Table SI8a.** Mulliken atomic charges for chlorpyrifos (CPF). Charges are presented in atomic units (e) (gas phase).

**Table SI8b.** Mulliken atomic charges for CPF-oxon. Charges are presented in atomic units (e) (gas phase).

| Atoms |  | Charge |
| --- | --- | --- |
| 0 | P | 0.569058 |
| 1 | O | -0.21034 |
| 2 | O | -0.29389 |
| 3 | C | -0.064376 |
| 4 | C | -0.295529 |
| 5 | C | -0.053785 |
| 6 | C | -0.259764 |
| 7 | S | 0.024968 |
| 8 | O | -0.331026 |
| 9 | C | 0.017129 |
| 10 | C | -0.184597 |
| 11 | C | 0.022281 |
| 12 | C | 0.126167 |
| 13 | C | 0.190645 |
| 14 | N | -0.181129 |
| 15 | C | -0.064717 |
| 16 | C | -0.083492 |
| 17 | C | -0.099952 |
| 18 | H | 0.125423 |
| 19 | H | 0.121751 |
| 20 | H | 0.118503 |
| 21 | H | 0.126511 |
| 22 | H | 0.119676 |
| 23 | H | 0.070808 |
| 24 | H | 0.043169 |
| 25 | H | 0.132435 |
| 26 | H | 0.08689 |
| 27 | H | 0.097686 |
| 28 | H | 0.129496 |

| Atoms | | Charge |
| --- | --- | --- |
| 0 | P | 0.832335 |
| 1 | O | -0.30822 |
| 2 | O | -0.287339 |
| 3 | C | -0.040421 |
| 4 | C | -0.30325 |
| 5 | C | -0.007065 |
| 6 | C | -0.331604 |
| 7 | O | -0.575781 |
| 8 | O | -0.327624 |
| 9 | C | 0.033178 |
| 10 | C | -0.1644 |
| 11 | C | 0.037496 |
| 12 | C | 0.142557 |
| 13 | C | 0.352528 |
| 14 | N | -0.192797 |
| 15 | C | -0.054201 |
| 16 | C | -0.060014 |
| 17 | C | -0.068637 |
| 18 | H | 0.115302 |
| 19 | H | 0.11871 |
| 20 | H | 0.108279 |
| 21 | H | 0.119756 |
| 22 | H | 0.120371 |
| 23 | H | 0.126289 |
| 24 | H | 0.105778 |
| 25 | H | 0.108047 |
| 26 | H | 0.106302 |
| 27 | H | 0.146824 |
| 28 | H | 0.1476 |

**Table SI9a.** NPA for chlorpyrifos (CPF). Charges are presented in atomic units (e)

**Table SI9b.** NPA for CPF-oxon. Charges are presented in atomic units (e)

| Atoms |  | Charge e |
| --- | --- | --- |
| 1 | P | 2.07326 |
| 2 | O | -0.80994 |
| 3 | O | -0.80225 |
| 4 | C | -0.06093 |
| 5 | C | -0.60529 |
| 6 | C | -0.06687 |
| 7 | C | -0.61642 |
| 8 | S | -0.58429 |
| 9 | O | -0.76754 |
| 10 | C | -0.11018 |
| 11 | C | -0.17697 |
| 12 | C | -0.10406 |
| 13 | C | 0.15617 |
| 14 | C | 0.47243 |
| 15 | N | -0.41676 |
| 16 | Cl | 0.04675 |
| 17 | Cl | 0.0568 |
| 18 | Cl | 0.05421 |
| 19 | H | 0.18123 |
| 20 | H | 0.18137 |
| 21 | H | 0.21151 |
| 22 | H | 0.21198 |
| 23 | H | 0.21239 |
| 24 | H | 0.19662 |
| 25 | H | 0.18362 |
| 26 | H | 0.21128 |
| 27 | H | 0.20671 |
| 28 | H | 0.22354 |
| 29 | H | 0.2416 |

| Atoms | | Charge e |
| --- | --- | --- |
| 1 | P | 2.57344 |
| 2 | O | -0.82161 |
| 3 | O | -0.8006 |
| 4 | C | -0.06075 |
| 5 | C | -0.60675 |
| 6 | C | -0.05675 |
| 7 | C | -0.61825 |
| 8 | O | -1.06835 |
| 9 | O | -0.74575 |
| 10 | C | -0.11577 |
| 11 | C | -0.17628 |
| 12 | C | -0.11404 |
| 13 | C | 0.16111 |
| 14 | C | 0.47981 |
| 15 | N | -0.44134 |
| 16 | Cl | 0.04406 |
| 17 | Cl | 0.05303 |
| 18 | Cl | 0.05111 |
| 19 | H | 0.18179 |
| 20 | H | 0.18237 |
| 21 | H | 0.2112 |
| 22 | H | 0.21266 |
| 23 | H | 0.2112 |
| 24 | H | 0.20487 |
| 25 | H | 0.18252 |
| 26 | H | 0.21301 |
| 27 | H | 0.20412 |
| 28 | H | 0.21838 |
| 29 | H | 0.24155 |

**Table SI10a**: Summary of Natural Population Analysis for the relHaant atoms in reactant, including their natural charges. core populations. valence populations. Rydberg contributions. and total populations (gas phase).

| Atom | Nº | Natural Charge (e) | Core | Valence | Rydberg | Total |
| --- | --- | --- | --- | --- | --- | --- |
| P | 1 | 1.27761 | 4.99891 | 1.14560 | 0.07788 | 6.22239 |
| O | 2 | -0.42994 | 0.99988 | 3.42193 | 0.00812 | 4.42994 |
| O | 3 | -0.40053 | 0.99982 | 3.38670 | 0.01401 | 4.40053 |
| C | 4 | -0.02195 | 0.99962 | 2.01393 | 0.00840 | 3.02195 |
| C | 5 | -0.31105 | 0.99972 | 2.30487 | 0.00645 | 3.31105 |
| C | 6 | -0.01534 | 0.99964 | 2.00670 | 0.00900 | 3.01534 |
| C | 7 | -0.32662 | 0.99972 | 2.32047 | 0.00643 | 3.32662 |
| O | 8 | -0.48213 | 0.99989 | 3.46231 | 0.01993 | 4.48213 |
| O | 9 | -0.33148 | 0.99986 | 3.31962 | 0.01200 | 4.33148 |
| C | 10 | 0.04483 | 0.99924 | 1.94062 | 0.01530 | 2.95517 |
| C | 11 | -0.11759 | 0.99944 | 2.10603 | 0.01211 | 3.11759 |
| C | 12 | 0.09876 | 0.99936 | 1.88544 | 0.01644 | 2.90124 |
| C | 13 | 0.16804 | 0.99945 | 1.81375 | 0.01875 | 2.83196 |
| C | 14 | 0.35366 | 0.99946 | 1.63151 | 0.01537 | 2.64634 |
| N | 15 | -0.23269 | 0.99958 | 2.71300 | 0.02011 | 3.73269 |
| Cl | 16 | 0.23805 | 4.99970 | 3.25369 | 0.00857 | 8.26195 |
| Cl | 17 | 0.27334 | 4.99969 | 3.21774 | 0.00923 | 8.22666 |
| Cl | 18 | 0.15274 | 4.99975 | 3.33749 | 0.01002 | 8.34726 |
| Nb | 19 | 0.81437 | 17.96920 | 1.70017 | 0.01627 | 19.68563 |
| O | 20 | -0.27170 | 0.99996 | 3.26470 | 0.00704 | 4.27170 |
| O | 21 | -0.27779 | 0.99996 | 3.26852 | 0.00931 | 4.27779 |
| H | 22 | 0.09438 | 0.00000 | 0.40468 | 0.00094 | 0.40562 |
| H | 23 | 0.10496 | 0.00000 | 0.39421 | 0.00084 | 0.39504 |
| H | 24 | 0.12647 | 0.00000 | 0.37317 | 0.00036 | 0.37353 |
| H | 25 | 0.10857 | 0.00000 | 0.39090 | 0.00053 | 0.39143 |
| H | 26 | 0.12005 | 0.00000 | 0.37950 | 0.00045 | 0.37995 |
| H | 27 | 0.11413 | 0.00000 | 0.38527 | 0.00061 | 0.38587 |
| H | 28 | 0.11308 | 0.00000 | 0.38630 | 0.00062 | 0.38692 |
| H | 29 | 0.13861 | 0.00000 | 0.36101 | 0.00038 | 0.36139 |
| H | 30 | 0.11582 | 0.00000 | 0.38355 | 0.00063 | 0.38418 |
| H | 31 | 0.11605 | 0.00000 | 0.38346 | 0.00049 | 0.38395 |
| H | 32 | 0.14527 | 0.00000 | 0.35387 | 0.00086 | 0.35473 |

**Table SI10b**: Summary of Natural Population Analysis for the relHaant atoms in TS, including their natural charges. core populations. valence populations. Rydberg contributions. and total populations (solvent).

| Atom | Nº | Natural Charge (e) | Core | Valence | Rydberg | Total |
| --- | --- | --- | --- | --- | --- | --- |
| P | 1 | 1.25699 | 4.99899 | 1.17063 | 0.07339 | 6.24301 |
| O | 2 | -0.39856 | 0.99985 | 3.38472 | 0.01400 | 4.39856 |
| O | 3 | -0.39259 | 0.99984 | 3.37901 | 0.01373 | 4.39259 |
| C | 4 | -0.01477 | 0.99963 | 2.00734 | 0.00780 | 3.01477 |
| C | 5 | -0.31544 | 0.99972 | 2.30890 | 0.00682 | 3.31544 |
| C | 6 | -0.01542 | 0.99963 | 2.00785 | 0.00794 | 3.01542 |
| C | 7 | -0.31858 | 0.99972 | 2.31247 | 0.00639 | 3.31858 |
| O | 8 | -0.45433 | 0.99990 | 3.43517 | 0.01926 | 4.45433 |
| O | 9 | -0.23585 | 0.99985 | 3.22759 | 0.00841 | 4.23585 |
| C | 10 | 0.07092 | 0.99926 | 1.91432 | 0.01549 | 2.92908 |
| C | 11 | -0.13737 | 0.99944 | 2.12544 | 0.01250 | 3.13737 |
| C | 12 | 0.13059 | 0.99935 | 1.85360 | 0.01646 | 2.86941 |
| C | 13 | 0.14082 | 0.99943 | 1.84143 | 0.01832 | 2.85918 |
| C | 14 | 0.32616 | 0.99949 | 1.65809 | 0.01625 | 2.67384 |
| N | 15 | -0.21206 | 0.99959 | 2.69195 | 0.02052 | 3.71206 |
| Cl | 16 | 0.16210 | 4.99974 | 3.32991 | 0.00825 | 8.33790 |
| Cl | 17 | 0.23720 | 4.99971 | 3.25406 | 0.00903 | 8.26280 |
| Cl | 18 | 0.09221 | 4.99979 | 3.39853 | 0.00948 | 8.40779 |
| Nb | 19 | 0.90023 | 17.96693 | 1.62181 | 0.01103 | 19.59977 |
| O | 20 | -0.31310 | 0.99996 | 3.30537 | 0.00777 | 4.31310 |
| O | 21 | -0.30235 | 0.99996 | 3.29492 | 0.00746 | 4.30235 |
| H | 22 | 0.10662 | 0.00000 | 0.39260 | 0.00077 | 0.39338 |
| H | 23 | 0.10513 | 0.00000 | 0.39420 | 0.00067 | 0.39487 |
| H | 24 | 0.12758 | 0.00000 | 0.37205 | 0.00037 | 0.37242 |
| H | 25 | 0.11507 | 0.00000 | 0.38439 | 0.00055 | 0.38493 |
| H | 26 | 0.11831 | 0.00000 | 0.38121 | 0.00048 | 0.38169 |
| H | 27 | 0.10440 | 0.00000 | 0.39493 | 0.00067 | 0.39560 |
| H | 28 | 0.11352 | 0.00000 | 0.38571 | 0.00077 | 0.38648 |
| H | 29 | 0.12965 | 0.00000 | 0.36999 | 0.00037 | 0.37035 |
| H | 30 | 0.11784 | 0.00000 | 0.38167 | 0.00049 | 0.38216 |
| H | 31 | 0.11449 | 0.00000 | 0.38502 | 0.00048 | 0.38551 |
| H | 32 | 0.14060 | 0.00000 | 0.35839 | 0.00101 | 0.35940 |

**Table SI10c**: Summary of Natural Population Analysis for the relHaant atoms in product, including their natural charges. core populations. valence populations. Rydberg contributions. and total populations (gas phase).

| Atom | Nº | Natural Charge (e) | Core | Valence | Rydberg | Total |
| --- | --- | --- | --- | --- | --- | --- |
| P | 1 | 1.24322 | 4.99871 | 1.18487 | 0.07320 | 6.25678 |
| O | 2 | -0.41417 | 0.99988 | 3.40561 | 0.00867 | 4.41417 |
| O | 3 | -0.37543 | 0.99983 | 3.36091 | 0.01468 | 4.37543 |
| C | 4 | -0.02722 | 0.99962 | 2.01898 | 0.00862 | 3.02722 |
| C | 5 | -0.31253 | 0.99972 | 2.30651 | 0.00629 | 3.31253 |
| C | 6 | -0.01733 | 0.99964 | 2.00829 | 0.00941 | 3.01733 |
| C | 7 | -0.32649 | 0.99972 | 2.32026 | 0.00652 | 3.32649 |
| O | 8 | -0.44509 | 0.99991 | 3.42444 | 0.02075 | 4.44509 |
| O | 9 | -0.30735 | 0.99986 | 3.29507 | 0.01242 | 4.30735 |
| C | 10 | 0.04983 | 0.99923 | 1.93482 | 0.01611 | 2.95017 |
| C | 11 | -0.13028 | 0.99944 | 2.11835 | 0.01249 | 3.13028 |
| C | 12 | 0.09784 | 0.99938 | 1.88613 | 0.01666 | 2.90216 |
| C | 13 | 0.16662 | 0.99947 | 1.81501 | 0.01890 | 2.83338 |
| C | 14 | 0.34923 | 0.99946 | 1.63471 | 0.01661 | 2.65077 |
| N | 15 | -0.23883 | 0.99958 | 2.71871 | 0.02054 | 3.73883 |
| Cl | 16 | 0.21053 | 4.99969 | 3.28085 | 0.00892 | 8.28947 |
| Cl | 17 | 0.25197 | 4.99969 | 3.23874 | 0.00961 | 8.24803 |
| Cl | 18 | 0.14420 | 4.99979 | 3.34700 | 0.00901 | 8.35580 |
| Nb | 19 | 0.82162 | 17.96664 | 1.69445 | 0.01729 | 19.67838 |
| O | 20 | -0.28144 | 0.99996 | 3.27408 | 0.00740 | 4.28144 |
| O | 21 | -0.28880 | 0.99996 | 3.27891 | 0.00994 | 4.28880 |
| H | 22 | 0.10533 | 0.00000 | 0.39367 | 0.00101 | 0.39467 |
| H | 23 | 0.11364 | 0.00000 | 0.38560 | 0.00076 | 0.38636 |
| H | 24 | 0.13052 | 0.00000 | 0.36913 | 0.00035 | 0.36948 |
| H | 25 | 0.10754 | 0.00000 | 0.39195 | 0.00051 | 0.39246 |
| H | 26 | 0.11951 | 0.00000 | 0.38005 | 0.00044 | 0.38049 |
| H | 27 | 0.11385 | 0.00000 | 0.38555 | 0.00060 | 0.38615 |
| H | 28 | 0.12169 | 0.00000 | 0.37769 | 0.00061 | 0.37831 |
| H | 29 | 0.14287 | 0.00000 | 0.35676 | 0.00037 | 0.35713 |
| H | 30 | 0.11913 | 0.00000 | 0.38033 | 0.00053 | 0.38087 |
| H | 31 | 0.11287 | 0.00000 | 0.38662 | 0.00051 | 0.38713 |
| H | 32 | 0.14295 | 0.00000 | 0.35613 | 0.00093 | 0.35705 |

**Table SI11a**: Summary of Natural Population Analysis for the relHaant atoms in reactant, including their natural charges. core populations. valence populations. Rydberg contributions. and total populations (solvent).

| Atom | Nº | Natural Charge (e) | Core | Valence | Rydberg | Total |
| --- | --- | --- | --- | --- | --- | --- |
| P | 1 | 1.26536 | 4.99897 | 1.16069 | 0.07498 | 6.23464 |
| O | 2 | -0.42813 | 0.99990 | 3.42018 | 0.00805 | 4.42813 |
| O | 3 | -0.40063 | 0.99983 | 3.38752 | 0.01328 | 4.40063 |
| C | 4 | -0.02226 | 0.99963 | 2.01414 | 0.00848 | 3.02226 |
| C | 5 | -0.31285 | 0.99973 | 2.30658 | 0.00653 | 3.31285 |
| C | 6 | -0.02119 | 0.99965 | 2.01233 | 0.00921 | 3.02119 |
| C | 7 | -0.32781 | 0.99972 | 2.32165 | 0.00644 | 3.32781 |
| O | 8 | -0.47309 | 0.99990 | 3.45383 | 0.01936 | 4.47309 |
| O | 9 | -0.32763 | 0.99986 | 3.31569 | 0.01207 | 4.32763 |
| C | 10 | 0.05024 | 0.99927 | 1.93535 | 0.01514 | 2.94976 |
| C | 11 | -0.12243 | 0.99945 | 2.11096 | 0.01202 | 3.12243 |
| C | 12 | 0.10342 | 0.99937 | 1.88102 | 0.01619 | 2.89658 |
| C | 13 | 0.17251 | 0.99946 | 1.80954 | 0.01849 | 2.82749 |
| C | 14 | 0.35625 | 0.99947 | 1.62894 | 0.01534 | 2.64375 |
| N | 15 | -0.23717 | 0.99958 | 2.71781 | 0.01977 | 3.73717 |
| Cl | 16 | 0.22810 | 4.99971 | 3.26386 | 0.00833 | 8.27190 |
| Cl | 17 | 0.26480 | 4.99970 | 3.22650 | 0.00899 | 8.23520 |
| Cl | 18 | 0.14561 | 4.99977 | 3.34505 | 0.00958 | 8.35439 |
| Nb | 19 | 0.83147 | 17.96860 | 1.68458 | 0.01536 | 19.66853 |
| O | 20 | -0.27266 | 0.99996 | 3.26564 | 0.00706 | 4.27266 |
| O | 21 | -0.27885 | 0.99996 | 3.26959 | 0.00930 | 4.27885 |
| H | 22 | 0.09749 | 0.00000 | 0.40162 | 0.00089 | 0.40251 |
| H | 23 | 0.10555 | 0.00000 | 0.39362 | 0.00083 | 0.39445 |
| H | 24 | 0.12807 | 0.00000 | 0.37158 | 0.00036 | 0.37193 |
| H | 25 | 0.10949 | 0.00000 | 0.38999 | 0.00052 | 0.39051 |
| H | 26 | 0.12032 | 0.00000 | 0.37923 | 0.00045 | 0.37968 |
| H | 27 | 0.11473 | 0.00000 | 0.38467 | 0.00059 | 0.38527 |
| H | 28 | 0.11382 | 0.00000 | 0.38557 | 0.00061 | 0.38618 |
| H | 29 | 0.13931 | 0.00000 | 0.36032 | 0.00037 | 0.36069 |
| H | 30 | 0.11615 | 0.00000 | 0.38322 | 0.00063 | 0.38385 |
| H | 31 | 0.11552 | 0.00000 | 0.38399 | 0.00049 | 0.38448 |
| H | 32 | 0.14650 | 0.00000 | 0.35265 | 0.00085 | 0.35350 |

**Table SI11b**: Summary of Natural Population Analysis for the relHaant atoms in TS, including their natural charges. core populations. valence populations. Rydberg contributions. and total populations (solvent).

| Atom | Nº | Natural Charge (e) | Core | Valence | Rydberg | Total |
| --- | --- | --- | --- | --- | --- | --- |
| P | 1 | 1.23559 | 4.99901 | 1.19405 | 0.07136 | 6.26441 |
| O | 2 | -0.42300 | 0.99991 | 3.41501 | 0.00807 | 4.42300 |
| O | 3 | -0.37964 | 0.99984 | 3.36534 | 0.01446 | 4.37964 |
| C | 4 | -0.02172 | 0.99962 | 2.01389 | 0.00821 | 3.02172 |
| C | 5 | -0.31323 | 0.99972 | 2.30688 | 0.00662 | 3.31323 |
| C | 6 | -0.01184 | 0.99964 | 2.00308 | 0.00912 | 3.01184 |
| C | 7 | -0.32711 | 0.99972 | 2.32080 | 0.00659 | 3.32711 |
| O | 8 | -0.44043 | 0.99991 | 3.42034 | 0.02017 | 4.44043 |
| O | 9 | -0.30204 | 0.99987 | 3.29064 | 0.01153 | 4.30204 |
| C | 10 | 0.05502 | 0.99923 | 1.93012 | 0.01564 | 2.94498 |
| C | 11 | -0.12760 | 0.99945 | 2.11588 | 0.01227 | 3.12760 |
| C | 12 | 0.10969 | 0.99936 | 1.87448 | 0.01647 | 2.89031 |
| C | 13 | 0.15846 | 0.99946 | 1.82346 | 0.01863 | 2.84154 |
| C | 14 | 0.33629 | 0.99946 | 1.64780 | 0.01645 | 2.66371 |
| N | 15 | -0.23162 | 0.99959 | 2.71147 | 0.02056 | 3.73162 |
| Cl | 16 | 0.20043 | 4.99971 | 3.29125 | 0.00861 | 8.29957 |
| Cl | 17 | 0.25472 | 4.99970 | 3.23638 | 0.00920 | 8.24528 |
| Cl | 18 | 0.15641 | 4.99974 | 3.33365 | 0.01020 | 8.34359 |
| Nb | 19 | 0.82159 | 17.96964 | 1.69285 | 0.01591 | 19.67841 |
| O | 20 | -0.28220 | 0.99996 | 3.27508 | 0.00716 | 4.28220 |
| O | 21 | -0.27763 | 0.99996 | 3.27030 | 0.00738 | 4.27763 |
| H | 22 | 0.10068 | 0.00000 | 0.39842 | 0.00090 | 0.39932 |
| H | 23 | 0.10755 | 0.00000 | 0.39160 | 0.00085 | 0.39245 |
| H | 24 | 0.12904 | 0.00000 | 0.37061 | 0.00036 | 0.37096 |
| H | 25 | 0.11251 | 0.00000 | 0.38698 | 0.00051 | 0.38749 |
| H | 26 | 0.12020 | 0.00000 | 0.37936 | 0.00044 | 0.37980 |
| H | 27 | 0.11045 | 0.00000 | 0.38892 | 0.00063 | 0.38955 |
| H | 28 | 0.11691 | 0.00000 | 0.38241 | 0.00068 | 0.38309 |
| H | 29 | 0.14080 | 0.00000 | 0.35883 | 0.00037 | 0.35920 |
| H | 30 | 0.11643 | 0.00000 | 0.38295 | 0.00062 | 0.38357 |
| H | 31 | 0.11235 | 0.00000 | 0.38716 | 0.00050 | 0.38765 |
| H | 32 | 0.14292 | 0.00000 | 0.35618 | 0.00090 | 0.35708 |

**Table SI11c**: Summary of Natural Population Analysis for the relHaant atoms in product, including their natural charges. core populations. valence populations. Rydberg contributions. and total populations (solvent).

| Atom | Nº | Natural Charge (e) | Core | Valence | Rydberg | Total |
| --- | --- | --- | --- | --- | --- | --- |
| P | 1 | 1.36937 | 4.99859 | 1.07795 | 0.05409 | 6.13063 |
| O | 2 | -0.32999 | 0.99986 | 3.31860 | 0.01153 | 4.32999 |
| O | 3 | -0.35989 | 0.99985 | 3.34766 | 0.01237 | 4.35989 |
| C | 4 | -0.00684 | 0.99963 | 1.99958 | 0.00764 | 3.00684 |
| C | 5 | -0.31387 | 0.99972 | 2.30751 | 0.00664 | 3.31387 |
| C | 6 | -0.02141 | 0.99963 | 2.01365 | 0.00813 | 3.02141 |
| C | 7 | -0.31348 | 0.99972 | 2.30763 | 0.00612 | 3.31348 |
| O | 8 | -0.15058 | 0.99997 | 3.14448 | 0.00613 | 4.15058 |
| O | 9 | -0.28099 | 0.99986 | 3.26727 | 0.01385 | 4.28099 |
| C | 10 | -0.00687 | 0.99925 | 1.99118 | 0.01643 | 3.00687 |
| C | 11 | -0.10370 | 0.99945 | 2.09229 | 0.01196 | 3.10370 |
| C | 12 | 0.00357 | 0.99927 | 1.98180 | 0.01536 | 2.99643 |
| C | 13 | 0.13319 | 0.99939 | 1.84915 | 0.01827 | 2.86681 |
| C | 14 | 0.29212 | 0.99941 | 1.69467 | 0.01380 | 2.70788 |
| N | 15 | -0.23298 | 0.99956 | 2.71464 | 0.01879 | 3.73298 |
| Cl | 16 | 0.14319 | 4.99974 | 3.34873 | 0.00833 | 8.35681 |
| Cl | 17 | 0.14323 | 4.99976 | 3.34816 | 0.00885 | 8.35677 |
| Cl | 18 | 0.12851 | 4.99977 | 3.36292 | 0.00879 | 8.37149 |
| H | 19 | 0.10714 | 0.00000 | 0.39214 | 0.00072 | 0.39286 |
| H | 20 | 0.10243 | 0.00000 | 0.39684 | 0.00072 | 0.39757 |
| H | 21 | 0.12694 | 0.00000 | 0.37268 | 0.00038 | 0.37306 |
| H | 22 | 0.11585 | 0.00000 | 0.38364 | 0.00050 | 0.38415 |
| H | 23 | 0.11631 | 0.00000 | 0.38319 | 0.00049 | 0.38369 |
| H | 24 | 0.09692 | 0.00000 | 0.40205 | 0.00103 | 0.40308 |
| H | 25 | 0.11365 | 0.00000 | 0.38559 | 0.00077 | 0.38635 |
| H | 26 | 0.12468 | 0.00000 | 0.37495 | 0.00037 | 0.37532 |
| H | 27 | 0.11730 | 0.00000 | 0.38225 | 0.00046 | 0.38270 |
| H | 28 | 0.11134 | 0.00000 | 0.38813 | 0.00053 | 0.38866 |
| H | 29 | 0.13570 | 0.00000 | 0.36341 | 0.00089 | 0.36430 |
| Nb | 30 | 0.94866 | 17.96850 | 1.57809 | 0.00475 | 19.55134 |
| O | 31 | -0.67278 | 0.99998 | 3.66718 | 0.00562 | 4.67278 |
| O | 32 | -0.13674 | 0.99997 | 3.13058 | 0.00619 | 4.13674 |

Table S112. Calculated energies and activation barrier for the alternative reaction pathway initiated by coordination of NbO₂²⁺ at the phosphoryl oxygen atom (P=O) of chlorpyrifos-oxon.

| Reaction pathway | Reactant Energy (Eh) | Transition State Energy (Eh) | ΔE‡ (Eh) | Ea (kcal·mol⁻¹) |
| --- | --- | --- | --- | --- |
| Coordination through the phosphoryl oxygen atom (P=O) | −2634.301148 | −2634.242515 | 0.058633 | 36.79 |

**REFERENCE:**

1. Tirado-Rives J, Jorgensen WL (2008) Performance of B3LYP density functional methods for a large set of organic molecules. J Chem Theory Comput 4:297–306. https://doi.org/10.1021/CT700248K/SUPPL_FILE/CT700248K-FILE002.PDF

2. Chai J Da, Head-Gordon M (2008) Long-range corrected hybrid density functionals with damped atom–atom dispersion corrections. Phys Chem Chem Phys 10:6615–6620. https://doi.org/10.1039/B810189B

3. Grimme S, Antony J, Ehrlich S, Krieg H (2010) A consistent and accurate ab initio parametrization of density functional dispersion correction (DFT-D) for the 94 elements H-Pu. J Chem Phys 132:. https://doi.org/10.1063/1.3382344/926936

4. Zhao Y, Truhlar DG (2008) The M06 suite of density functionals for main group thermochemistry, thermochemical kinetics, noncovalent interactions, excited states, and transition elements: Two new functionals and systematic testing of four M06-class functionals and 12 other functionals. Theor Chem Acc 120:215–241. https://doi.org/10.1007/S00214-007-0310-X/METRICS

5. Yanai T, Tew DP, Handy NC (2004) A new hybrid exchange-correlation functional using the Coulomb-attenuating method (CAM-B3LYP). Chem Phys Lett 393:51–57. https://doi.org/10.1016/j.cplett.2004.06.011
